# Dietary Intake of Riboflavin and Unsaturated Fatty Acid Can Improve the Multi-Domain Cognitive Function in Middle-Aged and Elderly Populations: A 2-Year Prospective Cohort Study

**DOI:** 10.3389/fnagi.2019.00226

**Published:** 2019-08-29

**Authors:** Lingwei Tao, Kuo Liu, Si Chen, Huiyan Yu, Yu An, Ying Wang, Xiaona Zhang, Yushan Wang, Zhongsheng Qin, Rong Xiao

**Affiliations:** ^1^School of Public Health, Capital Medical University, Beijing, China; ^2^Jincheng People’s Hospital, Jincheng, China

**Keywords:** cognitive impairment, dietary nutrients, riboflavin, unsaturated fatty acid, middle-aged and elderly people

## Abstract

**Objective:**

This study was aimed to explore the effects of dietary nutrients on cognitive function among the middle-aged and elderly populations.

**Methods:**

A prospective cohort study of 1,385 middle-aged and elderly people was conducted from January 2014 to December 2017. Dietary nutrients were assessed according to the food frequency questionnaire (FFQ) and China Food Composition Database (CFCD). Montreal cognitive assessment (MoCA) was used to evaluate the participants’ global cognitive function. Six other neuropsychological measures [auditory verbal learning test-immediate recall (AVLT-IR), auditory verbal learning test-short recall (AVLT-SR), auditory verbal learning test-long recall (AVLT-LR), logical memory test (LMT), digit span forward (DST-F), and digit span backward (DST-B)] were used to assess the verbal memory domain and the attention domain by principal component analysis (PCA). Multiple linear regressions were conducted to explore associations between nutrients and cognition. Sensitivity analyses were performed to confirm the results.

**Results:**

Dietary riboflavin was protective for global cognitive function (β = 1.31, 95% CI: 0.26, 2.35) and the verbal memory domain (β = 0.37, 95% CI: 0.02, 0.71). Unsaturated fatty acid (USFA) played a protective role in global cognitive function (β = 1.15, 95% CI: 0.16, 2.14). The protective effects of riboflavin and USFA on cognitive function were consistent and reliable when different confounders were adjusted during sensitivity analyses. During the follow-up, neuropsychological measure scores revealed a reduced decline in the high-riboflavin group (d-MoCA, *P* = 0.025; d-AVLT-IR, *P* = 0.001; d-DST-B, *P* = 0.004; and d-composite score, *P* = 0.004) and the high-USFA group (d-AVLT-IR, *P* = 0.007; d-LMT, *P* = 0.032; d-DST-B, *P* = 0.002; and d-composite score, *P* = 0.008).

**Conclusion:**

Higher intake of riboflavin and USFA can improve multi-dimensional cognitive functioning in middle-aged and elderly people. These findings were consistent in different models of sensitivity analyses.

## Introduction

Dementia and cognitive impairment are among the most common conditions that affect the aging population (Brodaty et al., 2014; Gheysen et al., 2018). By 2050, it is predicted that roughly 2 billion people will be aged 60 years or above, of which 131 million will be affected by dementia (Moore et al., 2018). Populations with MCI may develop AD at a rate of 10–15% per year compared with the general population at a rate of 1–2% per year (Geda et al., 2013; Janoutova et al., 2015). However, MCI, which is a transitional stage between normal aging and dementia, offers an opportunity for early detection and prevention of AD (Roberts et al., 2010; Timmers et al., 2018). Studies have shown that diet plays an important role in reducing the risk of dementia and cognitive impairment (Roberts et al., 2010; Sofi et al., 2010; Rijpma et al., 2017; Bruins et al., 2019). Our previous researches have demonstrated that diets rich in vegetables, eggs, marine products, nuts, and fruit can prevent cognitive impairment in the middle-aged and elderly populations (Zhao X. et al., 2015; Dong et al., 2016); however, it is necessary to identify the specific nutrients that are responsible for this. Some studies found that B vitamins, vitamin E, and polyunsaturated fatty acids (PUFAs) such as docosahexaenoic acid (DHA) and eicosapentaenoic acid (EPA) are beneficial for cognitive function in the elderly (Bruins et al., 2019). In a Singaporean study, researchers found that supplemental intake of omega-3 polyunsaturated fatty acids (*n* – 3 PUFA) can prevent cognitive impairment in the elderly (Gao et al., 2011). In a Japanese elderly population study, researchers found that that a low intake of carotene, vitamin B_2_, pantothenate, and calcium correlated with the development of cognitive impairment (Araki et al., 2017). However, the protective effects of nutrients on cognitive function were not consistent among different studies. Studies showed that supplementation of B vitamins was not beneficial for cognitive function, and supplementation of high-dose vitamin E most likely did not reduce the risk of progression to dementia (McCleery et al., 2018). Recent investigations of the therapeutic potential of supplementation or higher dietary intake of DHA in patients with AD have also produced inconsistent results (Bruins et al., 2019). Most of these studies did not use comprehensive neuropsychological measures to assess cognitive function in multiple cognitive domains. Besides that, most of the current studies are based on relatively small sample sizes. Moreover, the reason for the unstable results may partially be due to different strategies used to adjust for confounders. Thus, sensitivity analyses are needed to confirm positive results in different statistical models. The current study used a prospective cohort study, with a sample size of 1,385 in China from January 2014 to December 2017, and aimed to examine the potential associations between dietary nutrients and multi-domain cognitive function. Sensitivity analyses were used to confirm the positive results.

## Materials and Methods

### Study Population

**FIGURE 1:**
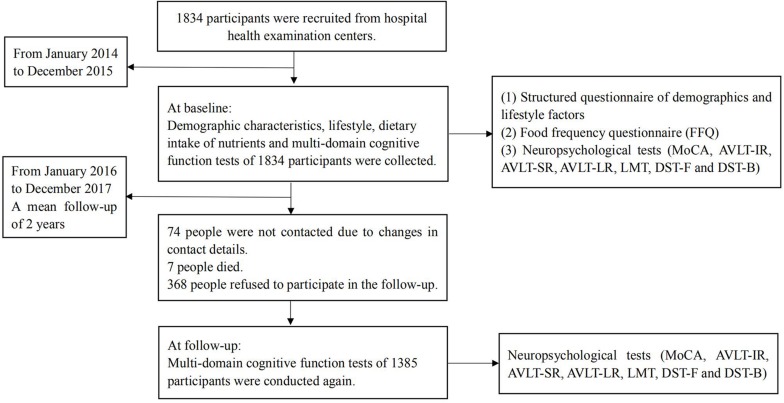
Flow chart representing the selection and follow-up of study participants.

The study participants were obtained from a longitudinal study, which aimed to investigate the effects of diet on cognitive function in the middle-aged and elderly populations (Rong et al., 2017). The participants were recruited from hospital health examination centers in China (including Jincheng People’s Hospital in Jincheng City of Shanxi Province and Linyi People’s Hospital in Linyi City in Shandong Province) from January 2014 to December 2017. Nurses from local hospital physical examination centers were trained to conduct questionnaires and cognitive function measures. The nurses who dealt with participants at baseline and follow-up were the same. A baseline survey was conducted in 1,834 participants from January 2014 to December 2015; moreover, demographic characteristics, dietary intake of nutrients, lifestyle factors, and multi-domain cognitive function measures of each participant were collected at this time. A follow-up survey was conducted from January 2016 to December 2017, with a mean follow-up of 2 years. The research team, once again, conducted multi-domain cognitive function measures for 1,385 participants during the follow-up process. During the follow-up, a total of 449 participants were lost; 74 participants did not inform us of their change of contact details; seven people died; and 368 people simply refused to participate in the follow-up. The flow chart outlining the selection and follow-up of study participants is shown in Figure 1. Participants were recruited according to the following inclusion criteria: 50–70 years old; not suffering from serious illnesses (history of heart or respiratory failure, renal failure, liver failure, cancer, and severe psychiatric disorders such as depression and schizophrenia); answering in the interview that they were capable of self-managing daily life; and voluntary participation in the study. Exclusion criteria included the following: middle-aged and elderly people who suffer from Parkinson’s disease (PD), dementia, or MCI; middle-aged and elderly people who currently use antidepressants and other medications for neurological diseases such as brain tumors, epilepsy, and sleep disorders; middle-aged and elderly people with conditions known to influence cognitive function (cerebral apoplexy and infarction, a recent history of alcohol abuse). The study was approved by the Ethics Committee of Capital Medical University (2013SY35) and conducted according to the Declaration of Helsinki. All participants provided written informed consent for their participation in the study.

### Demographic Characteristics

The investigators received a standardized training program before we performed the sample collection. A structured questionnaire was used to obtain data regarding demographics and lifestyle factors of participants by means of a face-to-face interview in local hospitals. Education was considered a categorical variable: low level (illiterate and elementary school), middle level (junior high school, senior high school, and technical secondary school), and high level (college and graduate school). The lifestyle factors included smoking (yes or no), drinking (yes or no), and reading (yes or no). A current smoker was defined as someone who smokes at least one cigarette per day for the past 6 months. A current drinker was defined as someone who consumes any type of alcoholic beverage at least once a week in the last 6 months (Wang et al., 2017). Reading was defined as someone who reads books/newspapers for >0 h per day. Height (m) was measured by using a height-measuring instrument; participants were asked to stand upright and remove their shoes (Zhang et al., 2017). Body weight (kg) was measured by using an automatic electronic scale; participants were asked to wear light clothing and remove footwear (Zhang et al., 2017). The BMI was calculated by weight (kg)/height^2^ (m^2^).

### Dietary Intake Assessment of Nutrients

The FFQ (Rong et al., 2017), which included 85 different types of specific foods (for example, rice, wheat, corn, tofu, soy milk, pork, beef, lamb, chicken, duck eggs, pure milk, yogurt, cabbage, potato, mushroom, peanut, walnut, etc.), was used to evaluate dietary intake. FFQ was used to assess the dietary intake of participants over the past year. The FFQ contained information including the consumption frequency (never, daily, weekly, monthly, or yearly) and the quantity of food consumed (volume or weight) (Lu et al., 2016). The quantity of food consumption was estimated with the help of food models (for example, measuring rulers, cups, and special charts). The FFQ was completed with the help of trained dietary investigators by using standardized language and unified instruction. The daily dietary intake levels of nutrients were calculated by using the CFCD (Yang et al., 2009). The good validity of FFQ was found in the intake of dietary nutrients, which were assessed by FFQ and 3-day food record (3DFR). Correlation coefficients of dietary nutrients, which were assessed by FFQ and 3DFR, ranged from 0.318 to 0.782. The good reliability of FFQ was found in the intake of dietary nutrients, which were assessed by applying FFQ twice. Correlation coefficients of dietary nutrients, which were assessed by applying FFQ twice, ranged from 0.379 to 0.740 (Huang et al., 2013).

### Multi-Domain Cognitive Function Assessment

Neuropsychological assessment was conducted at both baseline and follow-up. The comprehensive neuropsychological measures were used to assess the participants’ cognitive function. The measures were performed in a private room that had a quiet environment. In this study, the MoCA was used to evaluate global cognitive function (Chen et al., 2016). AVLT-IR was used to measure capability of immediate recall. AVLT-SR was used to measure capability of short recall. AVLT-LR was used to measure capability of delayed recall (Zhao Q. et al., 2015; Rong et al., 2017). LMT was a test from the Wechsler Memory Scale-Revised of China. The researchers read two stories aloud to the participants, one by one. The participants then attempted to restate the story immediately (Wang et al., 2016). Short-term memory and working memory were evaluated by DST-F and DST-B by using the Wechsler adult intelligence test-revised Chinese version (Sarnak et al., 2013; Lu et al., 2017). To evaluate the function of specific cognitive domains, principal component scores were constructed from the above six cognitive function measures (AVLT-IR, AVLT-SR, AVLT-LR, LMT, DST-F, and DST-B) by PCA. Therefore, global cognitive function was evaluated by MoCA scores. Specific cognitive domains (verbal memory domain and attention domain) were evaluated by principal component scores, which were calculated by PCA.

### Statistical Analyses

The data for continuous variables were reported as means ± standard deviation (SD) and categorical variables were presented as percentages (%). Independent-sample *t* test was used for continuous variables and chi-square test was used for categorical variables. Participants’ global cognitive function was measured by the MoCA score. Specific cognitive domains (verbal memory domain and attention domain) were evaluated by principal component scores, which were calculated by PCA. The type of rotation was varimax rotation. Principal component scores were constructed from the scores obtained from six cognitive function measures (AVLT-IR, AVLT-SR, AVLT-LR, LMT, DST-F, and DST-B) using PCA at baseline and at follow-up, respectively. Principal components were extracted when eigenvalue >1 (Sun and Xu, 2014). Subsequently, the principal component scores of cognitive function at follow-up were used as dependent variables in the multivariable linear regression models. The principal component scores of cognitive function at baseline were used as covariates in the multivariable linear regression models. The β values of nutrients calculated by multivariable linear regression represent an increment of dependent variable with each SD increment of nutrients. The multivariable linear regression model formula was as follows:

Y=follow–upβ+0βX1+baselineβX2+2βX3+3…+βXm+me

*Y*_follow–up_ represented the follow-up value pertaining to multi-domain cognitive function.

*X*_baseline_ represented the baseline value pertaining to multi-domain cognitive function.

*X*_2_…*X*_m_ represented different covariates, such as dietary nutrients, demographic characteristics, and lifestyle factors. As part of the sensitivity analysis, different models were built to further confirm the relationship between nutrients and cognitive function. In model 1, *X*_2_…*X*_m_ represented nutrients (protein, fat, carbohydrate, cholesterol, vitamin A, thiamin, riboflavin, niacin, vitamin C, vitamin E, saturated fatty acid, and USFA). In model 2, *X*_2_…*X*_m_ represented nutrients and demographic characteristics (sex, age, education, and BMI). In model 3, *X*_2_…*X*_m_ represented nutrients, demographic characteristics, and lifestyle (smoking, drinking, and reading).

Change of neuropsychological measure scores between follow-up and baseline was calculated as follows:

d-MoCA = follow-up MoCA score – baseline MoCA score;
d-AVLT-IR = follow-up AVLT-IR score – baseline AVLT-IR score;
d-AVLT-SR = follow-up AVLT-SR score at – baseline AVLT-SR score;
d-AVLT-LR = follow-up AVLT-LR score – baseline AVLT-LR score;
d-LMT = follow-up LMT score – baseline LMT score;
d-DST-F = follow-up DST-F score – baseline DST-F score;
d-DST-B = follow-up DST-B score – baseline DST-B score;
composite score = MoCA score + AVLT-IR score + AVLT-SR score + AVLT-LR score + LMT score + DST-F score + DST-B score;
d-composite score = follow-up composite score – baseline composite score.

All analyses were conducted using the IBM SPSS Statistics 24.0 software (SPSS, Chicago, IL, United States). A *P* value less than 0.05, in two sides, was considered to be statistically significant.

## Results

### Demographic Characteristics, Lifestyle Factors, Dietary Nutrients, and Neuropsychological Measures of 1,385 Middle-Aged and Elderly People at Baseline

The baseline characteristics data of 1,385 participants are shown in Table 1. The participants included 586 men (42.3%) and 799 women (57.7%). The average age was 58.75 years old and the average BMI was 24.68 kg/m^2^. Compared with the male group, the female group had a lower education level (*P* < 0.001), and a lower proportion of participants who smoked (*P* < 0.001), consumed alcohol (*P* < 0.001), and reported reading habits (*P* < 0.001). Females also had a lower dietary intake of nutrients [protein (*P* < 0.001), carbohydrate (*P* < 0.001), cholesterol (*P* = 0.001), thiamin (*P* < 0.001), niacin (*P* = 0.002), and saturated fatty acid (*P* = 0.024)]. The dietary intakes of fat, vitamin A, riboflavin, vitamin C, vitamin E, and USFA were not significantly different between the male and female groups. Compared with the male group, the female group had a higher baseline AVLT-IR score (*P* < 0.001), AVLT-SR score (*P* < 0.001), and AVLT-LR score (*P* < 0.001). In addition, the female group had a lower baseline MoCA score (*P* < 0.001), LMT score (*P* = 0.004), DST-F score (*P* = 0.001), and DST-B score (*P* = 0.019).

**TABLE 1:** Comparison of demographic characteristics, lifestyle, dietary nutrients, and multi-domain cognitive function of 1,385 middle-aged and elderly participants at baseline.

	**All *n* = 1385**	**Male *n* = 586**	**Female *n* = 799**	**χ ^2^ or *t* value**	***P* value**
Age (years)^a^	58.75 ± 4.43	59.10 ± 4.37	58.50 ± 4.47	2.47	0.013
Education *n* (%)^b,c^					
Low	230 (16.6)	49 (8.4)	181 (22.7)	78.57	<0.001
Middle	983 (71.0)	425 (72.5)	558 (69.8)		
High	172 (12.4)	112 (19.1)	60 (7.5)		
BMI (kg/m^2^)^a,d^	24.68 ± 2.92	25.05 ± 2.80	24.41 ± 2.97	4.05	<0.001
Smoking *n* (%)^b^					
No	1007 (72.7)	232 (39.6)	775 (97.0)	561.42	<0.001
Yes	378 (27.3)	354 (60.4)	24 (3.0)		
Drinking *n* (%)^b^					
No	979 (70.7)	228 (38.9)	751 (94.0)	495.05	<0.001
Yes	406 (29.3)	358 (61.1)	48 (6.0)		
Reading *n* (%)^b^					
No	857 (61.9)	293 (50.0)	564 (70.6)	60.75	<0.001
Yes	528 (38.1)	293 (50.0)	235 (29.4)		
Protein (g/d)^a^	56.97 ± 24.90	60.93 ± 26.97	54.06 ± 22.85	4.99	<0.001
Fat (g/d)^a^	62.21 ± 40.09	64.22 ± 43.70	60.73 ± 37.18	1.60	0.109
Carbohydrate (g/d)^a^	233.52 ± 96.97	250.51 ± 107.20	221.05 ± 86.70	5.47	<0.001
Cholesterol (mg/d)^a^	233.33 ± 153.59	249.16 ± 165.73	221.72 ± 143.03	3.30	0.001
Vitamin A (μg/d)^a^	684.72 ± 688.17	701.70 ± 736.84	672.27 ± 650.36	0.79	0.432
Thiamin (mg/d)^a^	1.11 ± 0.48	1.19 ± 0.52	1.06 ± 0.44	5.22	<0.001
Riboflavin (mg/d)^a^	1.24 ± 0.93	1.28 ± 0.99	1.21 ± 0.88	1.49	0.136
Niacin (mg/d)^a^	15.51 ± 9.60	16.43 ± 10.42	14.83 ± 8.90	3.07	0.002
Vitamin C (mg/d)^a^	108.74 ± 104.37	106.69 ± 107.72	110.23 ± 101.89	–0.62	0.533
Vitamin E (mg/d)^a^	40.13 ± 23.19	40.16 ± 23.84	40.12 ± 22.71	0.03	0.976
Saturated fatty acid (g/d)^a^	13.10 ± 7.54	13.63 ± 8.13	12.70 ± 7.06	2.26	0.024
USFA (g/d)^a,e^	43.08 ± 30.08	43.33 ± 32.05	42.89 ± 28.57	0.27	0.786
MoCA^a,f^	24.19 ± 3.26	24.72 ± 3.03	23.80 ± 3.37	5.34	<0.001
AVLT-IR^a,g^	15.62 ± 4.95	15.07 ± 4.64	16.02 ± 5.13	–3.59	<0.001
AVLT-SR^a,h^	5.50 ± 2.49	5.21 ± 2.38	5.71 ± 2.54	–3.78	<0.001
AVLT-LR^a,i^	4.63 ± 2.76	4.26 ± 2.53	4.89 ± 2.88	–4.32	<0.001
LMT^a,j^	10.54 ± 4.94	10.99 ± 4.92	10.21 ± 4.94	2.91	0.004
DST-F^a,k^	7.80 ± 1.16	7.92 ± 1.01	7.71 ± 1.25	3.49	0.001
DST-B^a,l^	4.27 ± 1.07	4.35 ± 1.04	4.21 ± 1.09	2.35	0.019

*^*a*^Continuous variables, means ± SD, and *t* value are presented; ^*b*^Categorical variables, numbers (percentages), and χ^2^ value are presented; ^*c*^Educational: low (illiterate and elementary school), middle (junior high school, senior high school, and technical secondary school), and high (college and graduate school); ^*d*^BMI, body mass index; ^*e*^USFA, unsaturated fatty acid; ^*f*^MoCA, Montreal cognitive assessment; ^*g*^AVLT-IR, auditory verbal learning test-immediate recall; ^*h*^AVLT-SR, auditory verbal learning test-short recall; ^*i*^AVLT-LR, auditory verbal learning test-long recall; ^*j*^LMT, logical memory test; ^*k*^DST-F, digit span test-forward; ^*l*^DST-B, digit span test-backward.*

### Principal Components Defined by PCA at Baseline and Follow-Up

This study used PCA to extract principal components from the baseline scores of six cognitive function measures (AVLT-IR, AVLT-SR, AVLT-LR, LMT, DST-F, and DST-B). Results of PCA revealed that the KMO value was 0.77 and the Bartlett sphericity test value was 3865.37 (df = 15, *P* < 0.001). The results showed that the data were suitable for PCA (Mills et al., 2010). Principal component extraction was carried out under the condition of undefined principal component number. Two principal components (Eigenvalue > 1) were extracted and the cumulative variance contribution rate was 70.25%. The first principal component was primarily composed of AVLT-IR, AVLT-SR, and AVLT-LR, which reflected the verbal memory domain. The second principal component was primarily composed of LMT, DST-F, and DST-B, which reflected the attention domain (Table 2).

**TABLE 2:** Rotated component matrix, eigenvalue, and cumulative variance contribution rate of the scores for multi-domain cognitive function measures among 1385 middle-aged and elderly participants at baseline and at follow-up.

		**Baseline**			**Follow-up**	
	**Measures**	**Principal component 1^a^**	**Principal component 2^b^**	**Measures**	**Principal component 1^a^**	**Principal component 2^b^**
	AVLT-IR^c^	0.86	0.17	AVLT-IR^c^	0.86	0.23
	AVLT-SR^d^	0.94	0.11	AVLT-SR^d^	0.92	0.16
	AVLT-LR^e^	0.93	0.13	AVLT-LR^e^	0.91	0.15
	LMT^f^	0.36	0.56	LMT^f^	0.41	0.58
	DST-F^g^	−0.09	0.82	DST-F^g^	0.05	0.79
	DST-B^h^	0.20	0.71	DST-B^h^	0.15	0.69
Eigenvalue		2.67	1.55		2.61	1.55
Variance contribution rate (%)		44.48	25.77		43.48	25.78
Cumulative variance contribution rate (%)		44.48	70.25		43.48	69.26
Naming of cognitive function domains		Verbal memory domain	Attention domain		Verbal memory domain	Attention domain

*^*a*^Principal component 1, verbal memory domain; ^*b*^Principal component 2, attention domain; ^*c*^AVLT-IR, auditory verbal learning test-immediate recall; ^*d*^AVLT-SR, auditory verbal learning test-short recall; ^*e*^AVLT-LR, auditory verbal learning test-long recall; ^*f*^LMT, logical memory test; ^*g*^DST-F, digit span test-forward; ^*h*^DST-B, digit span test-backward.*

Principal component analysis was also used to extract principal components from the follow-up scores of six cognitive function measures. The KMO value was 0.80 and the Bartlett sphericity test value was 3553.40 (df = 15, *P* < 0.001). Two principal components (Eigenvalue > 1) were extracted. The cumulative variance contribution rate was 69.26%. The first principal component was also primarily composed of AVLT-IR, AVLT-SR, and AVLT-LR, and was also interpreted to reflect the verbal memory domain. The second principal component was also primarily composed of LMT, DST-F, and DST-B, and was also interpreted to reflect the attention domain. Details are shown in Table 2.

### Associations Between Nutrients and Multi-Domain Cognitive Function

After adjusting for the baseline score pertaining to cognitive function (MoCA, verbal memory, and attention domain), nutrients (protein, fat, carbohydrate, cholesterol, vitamin A, thiamin, riboflavin, niacin, vitamin C, vitamin E, saturated fatty acid, and USFA), demographic characteristics (sex, age, education, and BMI), and lifestyle (smoking, drinking, and reading), the results of multivariable linear regression revealed that dietary intake of riboflavin was a protective factor in the global cognitive function (β = 1.31, 95% CI: 0.26, 2.35) and the verbal memory domain (β = 0.37, 95% CI: 0.02, 0.71). Dietary intake of USFA was also a protective factor in the global cognitive function (β = 1.15, 95% CI: 0.16, 2.14). Details are shown in Table 3.

**TABLE 3:** Relationship of nutrients, demographic characteristics, and lifestyle factors to multi-domain cognitive function among 1385 middle-aged and elderly participants.

	**Global cognitive function of follow-up**			**Verbal memory of follow-up**			**Attention of follow-up**		
	**β (95% CI)^a^**	***t***	***P* value**	**β (95% CI)^a^**	***t***	***P* value**	**β (95% CI)^a^**	***t***	***P* value**
MoCA of baseline^b^	0.49(0.44,0.53)	20.52	<0.001	−	−	−	−	−	−
Verbal memory of baseline	−	−	−	0.47(0.42,0.52)	19.53	<0.001	−	−	−
Attention of baseline	−	−	−	−	−	−	0.41(0.36,0.46)	16.67	<0.001
Protein (g/day)	−0.41(−1.21,0.39)	–1.01	0.311	−0.15(−0.41,0.12)	–1.10	0.273	−0.03(−0.31,0.24)	–0.24	0.808
Fat (g/day)	−0.71(−1.48,0.05)	–1.83	0.067	−0.18(−0.44,0.07)	–1.42	0.155	−0.02(−0.28,0.24)	–0.13	0.898
Carbohydrate (g/day)	0.19(−0.20,0.57)	0.95	0.344	−0.06(−0.19,0.06)	–0.98	0.328	0.09(−0.04,0.22)	1.35	0.176
Cholesterol (mg/day)	0.02(−0.26,0.29)	0.14	0.887	0.09(−0.002,0.18)	1.93	0.054	0.02(−0.08,0.11)	0.37	0.714
Vitamin A (μg/day)	−0.70(−1.79,0.38)	–1.28	0.202	−0.32(−0.68,0.04)	–1.74	0.082	0.04(−0.33,0.41)	0.23	0.818
Thiamin (mg/day)	−0.04(−0.59,0.50)	–0.15	0.881	0.08(−0.10,0.26)	0.84	0.403	−0.10(−0.29,0.08)	–1.07	0.287
Riboflavin (mg/day)	1.31(0.26,2.35)	2.45	0.015	0.37(0.02,0.71)	2.08	0.038	−0.11(−0.46,0.25)	–0.60	0.549
Niacin (mg/day)	0.22(−0.40,0.83)	0.69	0.490	0.08(−0.12,0.29)	0.80	0.426	0.03(−0.18,0.24)	0.30	0.766
Vitamin C (mg/day)	−0.36(−0.86,0.13)	–1.44	0.151	−0.01(−0.18,0.15)	–0.17	0.868	0.12(−0.05,0.29)	1.42	0.155
Vitamin E (mg/day)	−0.46(−0.97,0.05)	–1.76	0.079	0.02(−0.15,0.18)	0.18	0.858	−0.11(−0.28,0.06)	–1.25	0.210
Saturated fatty acid (g/day)	−0.08(−0.62,0.46)	–0.29	0.772	−0.13(−0.31,0.05)	–1.47	0.142	0.06(−0.13,0.24)	0.61	0.542
USFA (g/day)^c^	1.15(0.16,2.14)	2.29	0.022	0.26(−0.07,0.58)	1.54	0.125	0.12(−0.22,0.45)	0.67	0.505
Sex (Female)	0.12(−0.28,0.52)	0.57	0.569	0.16(0.02,0.29)	2.29	0.022	−0.06(−0.20,0.08)	–0.85	0.395
Age (years)	−0.03(−0.06,0.004)	–1.69	0.092	−0.03(−0.04,−0.02)	–4.68	<0.001	0.01(−0.001,0.02)	1.85	0.065
Education^d^									
Low	Ref.	−	−	Ref.	−	−	Ref.	−	−
Middle	0.82(0.41,1.22)	3.97	<0.001	0.07(−0.06,0.20)	1.10	0.272	0.36(0.23,0.50)	5.33	<0.001
High	1.57(0.99,2.15)	5.27	<0.001	0.13(−0.06,0.32)	1.36	0.173	0.62(0.43,0.82)	6.23	<0.001
BMI (kg/m^2^)^e^	−0.002(−0.049,0.046)	–0.06	0.950	−0.02(−0.04,−0.003)	–2.37	0.018	−0.01(−0.02,0.01)	–0.94	0.346
Smoking (Yes)	−0.07(−0.49,0.35)	–0.34	0.735	0.01(−0.13,0.15)	0.15	0.883	0.03(−0.12,0.17)	0.37	0.709
Drinking (Yes)	0.11(−0.28,0.50)	0.56	0.579	0.01(−0.12,0.14)	0.08	0.939	−0.04(−0.17,0.09)	–0.59	0.555
Reading (Yes)	0.39(0.08,0.70)	2.50	0.013	0.15(0.05,0.26)	3.01	0.003	0.06(−0.04,0.17)	1.17	0.242

*^*a*^β values of nutrients represent an increment of each SD increment; ^*b*^MoCA, Montreal cognitive assessment; ^*c*^USFA, unsaturated fatty acid; ^*d*^Educational: low (illiterate and elementary school), middle (junior high school, senior high school, and technical secondary school), and high (college and graduate school); ^*e*^BMI, body mass index.*

With the same adjustment as above, the results of multivariable linear regression also showed that being female played a protective role in the verbal memory domain (β = 0.16, 95% CI: 0.02, 0.29). Reading was a protective factor in the global cognitive function (β = 0.39, 95% CI: 0.08, 0.70) and the verbal memory domain (β = 0.15, 95% CI: 0.05, 0.26). A high education level also played a protective role in the global cognitive function (β = 1.57, 95% CI: 0.99, 2.15) and the attention domain (β = 0.62, 95% CI: 0.43, 0.82). Age (β = –0.03, 95% CI: –0.04, –0.02) and BMI (β = –0.02, 95% CI: –0.04, –0.003) were risk factors for the verbal memory domain (Table 3).

### Sensitivity Analysis of the Association Between Riboflavin, USFA, and Multi-Domain Cognitive Function in Different Models

**FIGURE 2:**
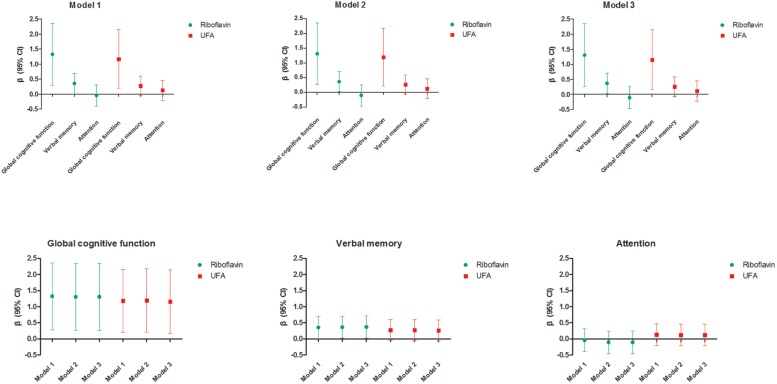
Sensitivity analysis of the association between riboflavin, USFA, and multi-domain cognitive function in different models. Model 1 adjusted for baseline value of multi-domain cognitive function (MoCA of baseline, verbal memory of baseline, or attention of baseline) and other nutrients (protein, fat, carbohydrate, cholesterol, vitamin A, thiamin, niacin, vitamin C, vitamin E, and saturated fatty acid). Model 2 adjusted for baseline value of multi-domain cognitive function (MoCA of baseline, verbal memory of baseline, or attention of baseline), other nutrients (protein, fat, carbohydrate, cholesterol, vitamin A, thiamin, niacin, vitamin C, vitamin E, and saturated fatty acid), and demographic characteristics (sex, age, education, and BMI). Model 3 adjusted for baseline value of multi-domain cognitive function (MoCA of baseline, verbal memory of baseline, or attention of baseline), other nutrients (protein, fat, carbohydrate, cholesterol, vitamin A, thiamin, niacin, vitamin C, vitamin E, and saturated fatty acid), demographic characteristics (sex, age, education, and BMI), and lifestyle (smoking, drinking, and reading). β values of nutrients represent an increment of one unit (each SD increment). USFA, unsaturated fatty acid; MoCA, Montreal cognitive assessment; BMI, body mass index.

Riboflavin was significantly associated with global cognitive function (β = 1.32, 95% CI: 0.28, 2.36) in model 1. The association was consistent when further adjusted for other covariates in model 2 and model 3 (β = 1.31, 95% CI: 0.26, 2.35 and β = 1.31, 95% CI: 0.26, 2.35). Riboflavin was also positively associated with the verbal memory domain (β = 0.35, 95% CI: 0.01, 0.70) in model 1. The association remained significant when it further adjusted for other covariates in model 2 and model 3 (β = 0.36, 95% CI: 0.02, 0.71 and β = 0.37, 95% CI: 0.02, 0.71). Furthermore, USFA was significantly associated with global cognitive function in model 1, model 2, and model 3 (β = 1.18, 95% CI: 0.19, 2.16; β = 1.19, 95% CI: 0.21, 2.18 and β = 1.15, 95% CI: 0.16, 2.14). The results of sensitivity analyses were consistent and reliable in different models (Table 4 and Figure 2).

**TABLE 4:** Sensitivity analysis of the association between riboflavin, USFA^a^, and multi-domain cognitive function in different models.

	**Global cognitive function of follow-up**				**Verbal memory of follow-up**				**Attention of follow-up**			
	**Model**	**β (95% CI)^b^**	***t***	***P* value**	**Model**	**β (95% CI)^b^**	***t***	***P* value**	**Model**	**β (95% CI)^b^**	***t***	***P* value**
Riboflavin (mg/day)	Model 1^c^	1.32 (0.28, 2.36)	2.50	0.013	Model 1^c^	0.35 (0.01, 0.70)	2.02	0.043	Model 1^c^	−0.04 (−0.40, 0.31)	−0.24	0.811
	Model 2^d^	1.31 (0.26, 2.35)	2.46	0.014	Model 2^d^	0.36 (0.02, 0.71)	2.05	0.040	Model 2^d^	−0.11 (−0.46, 0.24)	−0.62	0.539
	Model 3^e^	1.31 (0.26, 2.35)	2.45	0.015	Model 3^e^	0.37 (0.02, 0.71)	2.08	0.038	Model 3^e^	−0.11 (−0.46, 0.25)	−0.60	0.549
USFA (g/day)^*a*^	Model 1^c^	1.18 (0.19, 2.16)	2.35	0.019	Model 1^c^	0.28 (−0.05, 0.60)	1.66	0.097	Model 1^c^	0.13 (−0.21, 0.46)	0.75	0.455
	Model 2^d^	1.19 (0.21, 2.18)	2.38	0.018	Model 2^d^	0.27 (−0.06, 0.59)	1.60	0.109	Model 2^d^	0.12 (−0.21, 0.46)	0.71	0.480
	Model 3^e^	1.15 (0.16, 2.14)	2.29	0.022	Model 3^e^	0.26 (−0.07, 0.58)	1.54	0.125	Model 3^e^	0.12 (−0.22, 0.45)	0.67	0.505

*^*a*^USFA, unsaturated fatty acid; ^*b*^β values of nutrients represent an increment of one unit (each SD increment); ^*c*^Model 1 adjusted for baseline value of multi-domain cognitive function [Montreal cognitive assessment (MoCA) of baseline, verbal memory of baseline, or attention of baseline] and other nutrients (protein, fat, carbohydrate, cholesterol, vitamin A, thiamin, niacin, vitamin C, vitamin E, and saturated fatty acid); ^*d*^Model 2 adjusted for baseline value of multi-domain cognitive function (MoCA of baseline, verbal memory of baseline or attention of baseline), other nutrients (protein, fat, carbohydrate, cholesterol, vitamin A, thiamin, niacin, vitamin C, vitamin E, and saturated fatty acid), and demographic characteristics [sex, age, education, and body mass index (BMI)]; ^*e*^Model 3 adjusted for baseline value of multi-domain cognitive function (MoCA of baseline, verbal memory of baseline or attention of baseline), other nutrients (protein, fat, carbohydrate, cholesterol, vitamin A, thiamin, niacin, vitamin C, vitamin E, and saturated fatty acid), demographic characteristics (sex, age, education, and BMI), and lifestyle (smoking, drinking, and reading).*

### Comparison of the Differences of Neuropsychological Measure Scores Between Follow-Up and Baseline in the Low-/High-Riboflavin Groups and the Low-/High-USFA Groups

According to the mean intake of dietary riboflavin, the participants were divided into a low-riboflavin group (≤1.24 mg/day) and a high-riboflavin group (>1.24 mg/day). Compared with the low-riboflavin group, the d-MoCA, d-AVLT-IR, and d-DST-B of the high-riboflavin group were higher (*P* = 0.025, *P* = 0.001, and *P* = 0.004, respectively). The d-composite score of the high-riboflavin group was also higher (*P* = 0.004). According to the mean intake of dietary USFA, the participants were divided into a low-USFA group (≤43.08 g/day) and a high-USFA group (>43.08 g/day). Compared with the low-USFA group, the d-AVLT-IR, d-LMT, and d-DST-B of the high-USFA group were higher (*P* = 0.007, *P* = 0.032, and *P* = 0.002, respectively). The d-composite score of the high-USFA group was also higher (*P* = 0.008). Details are shown in Table 5. In females, compared with the low-riboflavin group, the d-AVLT-IR and d-DST-B of the high-riboflavin group were higher (*P* = 0.007 and *P* = 0.006, respectively). In females, the d-composite score of the high-riboflavin group was also higher (*P* = 0.036). In females, compared with the low-USFA group, the d-AVLT-IR, d-LMT, and d-DST-B of the high-USFA group were higher (*P* = 0.016, *P* = 0.020, and *P* < 0.001, respectively). In females, the d-composite score of the high-USFA group was also higher (*P* = 0.003). In males, compared with the low-riboflavin group, the d-composite score of the high-riboflavin group was higher (*P* = 0.035). Comparisons of the differences of neuropsychological measure scores between the low-USFA group and the high-USFA group were not statistically significant. Details are shown in Tables 6, 7.

**TABLE 5:** Comparisons of the differences of neuropsychological measure scores between follow-up and baseline in the low-/high-riboflavin groups and the low-/high-USFA groups.

	**Riboflavin groups (mg/day)**				**USFA groups (g/day)**			
	**Low group (≤1.24 mg/day)** ***n* = 887**	**High group (>1.24 mg/day)** ***n* = 498**	***t* value**	***P* value**	**Low group (≤43.08 g/day)** ***n* = 852**	**High group (>43.08 g/day)** ***n* = 533**	***t* value**	***P* value**
d-MoCA^a,b^	−0.17 ± 0.10	0.20 ± 0.13	−2.24	0.025	−0.07 ± 0.11	0.02 ± 0.12	−0.54	0.591
d-AVLT-IR^a,c^	−1.07 ± 0.17	−0.13 ± 0.24	−3.31	0.001	−1.03 ± 0.18	−0.26 ± 0.22	−2.72	0.007
d-AVLT-SR^a,d^	−0.35 ± 0.08	−0.23 ± 0.11	−0.86	0.393	−0.33 ± 0.08	−0.27 ± 0.11	−0.49	0.623
d-AVLT-LR^a,e^	−0.50 ± 0.09	−0.27 ± 0.12	−1.56	0.118	−0.45 ± 0.09	−0.37 ± 0.11	−0.58	0.565
d-LMT^a,f^	−0.58 ± 0.16	−0.37 ± 0.20	−0.81	0.421	−0.72 ± 0.16	−0.16 ± 0.20	−2.15	0.032
d-DST-F^a,g^	−0.07 ± 0.05	−0.21 ± 0.06	1.84	0.066	−0.11 ± 0.05	−0.14 ± 0.06	0.45	0.654
d-DST-B^a,h^	−0.47 ± 0.05	−0.25 ± 0.06	−2.88	0.004	−0.48 ± 0.05	−0.25 ± 0.06	−3.18	0.002
d-composite score^a,i^	−3.23 ± 0.40	−1.26 ± 0.55	−2.92	0.004	−3.20 ± 0.42	−1.44 ± 0.51	−2.66	0.008

*^*a*^Continuous variables, means ± SE, and *t* value are presented; ^*b*^d-MoCA = follow-up MoCA score - baseline MoCA score; ^*c*^d-AVLT-IR = follow-up AVLT-IR score - baseline AVLT-IR score; ^*d*^d-AVLT-SR = follow-up AVLT-SR score - baseline AVLT-SR score; ^*e*^d-AVLT-LR = follow-up AVLT-LR score - baseline AVLT-LR score; ^*f*^d-LMT = follow-up LMT score - baseline LMT score; ^*g*^d-DST-F = follow-up DST-F score - baseline DST-F score; ^*h*^d-DST-B = follow-up DST-B score - baseline DST-B score; ^*i*^d-composite score = follow-up composite score - baseline composite score. USFA, unsaturated fatty acid; MoCA, Montreal cognitive assessment; AVLT-IR, auditory verbal learning test-immediate recall; AVLT-SR, auditory verbal learning test-short recall; AVLT-LR, auditory verbal learning test-long recall; LMT, logical memory test; DST-F, digit span test-forward; DST-B, digit span test-backward.*

**TABLE 6:** Comparisons of the differences of neuropsychological measure scores between follow-up and baseline in the low-/high-riboflavin groups and the low-/high-USFA groups among the male population.

	**Riboflavin groups (mg/day)**				**USFA groups (g/day)**			
	**Low group (≤1.28 mg/day)** ***n* = 392**	**High group (>1.28 mg/day)** ***n* = 194**	***t* value**	***P* value**	**Low group (≤43.33 g/day)** ***n* = 359**	**High group (>43.33 g/day)** ***n* = 227**	***t* value**	***P* value**
d-MoCA^a,b^	−0.37 ± 0.15	0.10 ± 0.19	−1.83	0.068	−0.19 ± 0.16	−0.25 ± 0.18	0.25	0.803
d-AVLT-IR^a,c^	−0.97 ± 0.24	−0.18 ± 0.40	−1.78	0.076	−0.93 ± 0.27	−0.36 ± 0.35	−1.32	0.186
d-AVLT-SR^a,d^	−0.30 ± 0.12	−0.13 ± 0.18	−0.78	0.437	−0.25 ± 0.12	−0.23 ± 0.17	−0.08	0.933
d-AVLT-LR^a,e^	−0.48 ± 0.13	−0.30 ± 0.20	−0.78	0.436	−0.38 ± 0.14	−0.49 ± 0.17	0.50	0.617
d-LMT^a,f^	−0.74 ± 0.25	−0.27 ± 0.34	−1.11	0.270	−0.68 ± 0.26	−0.44 ± 0.32	−0.59	0.558
d-DST-F^a,g^	−0.11 ± 0.07	−0.19 ± 0.09	0.68	0.495	−0.14 ± 0.07	−0.13 ± 0.08	−0.15	0.878
d-DST-B^a,h^	−0.46 ± 0.07	−0.25 ± 0.09	−1.87	0.062	−0.41 ± 0.07	−0.35 ± 0.08	−0.59	0.554
d-composite score^a,i^	−3.43 ± 0.59	−1.22 ± 0.89	−2.11	0.035	−2.99 ± 0.62	−2.25 ± 0.82	−0.73	0.465

*^*a*^Continuous variables, means ± SE, and *t* value are presented; ^*b*^d-MoCA = follow-up MoCA score - baseline MoCA score; ^*c*^d-AVLT-IR = follow-up AVLT-IR score - baseline AVLT-IR score; ^*d*^d-AVLT-SR = follow-up AVLT-SR score - baseline AVLT-SR score; ^*e*^d-AVLT-LR = follow-up AVLT-LR score - baseline AVLT-LR score; ^*f*^d-LMT = follow-up LMT score - baseline LMT score; ^*g*^d-DST-F = follow-up DST-F score - baseline DST-F score; ^*h*^d-DST-B = follow-up DST-B score - baseline DST-B score; ^*i*^d-composite score = follow-up composite score - baseline composite score. USFA, unsaturated fatty acid; MoCA, Montreal cognitive assessment; AVLT-IR, auditory verbal learning test-immediate recall; AVLT-SR, auditory verbal learning test-short recall; AVLT-LR, auditory verbal learning test-long recall; LMT, logical memory test; DST-F, digit span test-forward; DST-B, digit span test-backward.*

**TABLE 7:** Comparisons of the differences of neuropsychological measure scores between follow-up and baseline in the low-/high-riboflavin groups and the low-/high-USFA groups among the female population.

	**Riboflavin groups (mg/day)**				**USFA groups (g/day)**			
	**Low group (≤1.21 mg/day)** ***n* = 496**	**High group (>1.21 mg/day)** ***n* = 303**	***t* value**	***P* value**	**Low group (≤42.89 g/day)** ***n* = 493**	**High group (>42.89 g/day)** ***n* = 306**	***t* value**	***P* value**
d-MoCA^a,b^	−0.06 ± 0.14	0.33 ± 0.17	−1.76	0.080	−0.01 ± 0.14	0.25 ± 0.17	−1.20	0.232
d-AVLT-IR^a,c^	−1.14 ± 0.23	−0.13 ± 0.29	−2.69	0.007	−1.10 ± 0.24	−0.20 ± 0.28	−2.42	0.016
d-AVLT-SR^a,d^	−0.39 ± 0.11	−0.31 ± 0.13	−0.45	0.653	−0.40 ± 0.11	−0.29 ± 0.13	−0.64	0.526
d-AVLT-LR^a,e^	−0.53 ± 0.12	−0.23 ± 0.15	−1.56	0.119	−0.50 ± 0.12	−0.28 ± 0.16	−1.10	0.272
d-LMT^a,f^	−0.43 ± 0.21	−0.48 ± 0.26	0.14	0.885	−0.74 ± 0.21	0.02 ± 0.24	−2.32	0.020
d-DST-F^a,g^	−0.04 ± 0.07	−0.21 ± 0.07	1.64	0.101	−0.09 ± 0.07	−0.14 ± 0.08	0.54	0.591
d-DST-B^a,h^	−0.50 ± 0.07	−0.21 ± 0.08	−2.75	0.006	−0.53 ± 0.07	−0.17 ± 0.08	−3.58	<0.001
d-composite score^a,i^	−3.09 ± 0.54	−1.24 ± 0.69	−2.10	0.036	−3.37 ± 0.56	−0.81 ± 0.64	−3.00	0.003

*^*a*^Continuous variables, means ± SE, and *t* value are presented; ^*b*^d-MoCA = follow-up MoCA score - baseline MoCA score; ^*c*^d-AVLT-IR = follow-up AVLT-IR score - baseline AVLT-IR score; ^*d*^d-AVLT-SR = follow-up AVLT-SR score - baseline AVLT-SR score; ^*e*^d-AVLT-LR = follow-up AVLT-LR score - baseline AVLT-LR score; ^*f*^d-LMT = follow-up LMT score - baseline LMT score; ^*g*^d-DST-F = follow-up DST-F score - baseline DST-F score; ^*h*^d-DST-B = follow-up DST-B score - baseline DST-B score; ^*i*^d-composite score = follow-up composite score - baseline composite score. USFA, unsaturated fatty acid; MoCA, Montreal cognitive assessment; AVLT-IR, auditory verbal learning test-immediate recall; AVLT-SR, auditory verbal learning test-short recall; AVLT-LR, auditory verbal learning test-long recall; LMT, logical memory test; DST-F, digit span test-forward; DST-B, digit span test-backward.*

## Discussion

Epidemiological evidence supports the idea that certain dietary factors play an important role in brain health; this presents us with new strategies to prevent dementia (Moore et al., 2018). Poor nutrition is recognized as a modifiable risk factor in the development of cognitive impairment in the aging population; moreover, improved nutrition may prevent or delay the onset of cognitive impairment (Lu et al., 2016; Porter et al., 2016). There is a wealth of literature indicating that diet can exert profound effects on cognitive function (Roberts et al., 2010; Sofi et al., 2010; Rijpma et al., 2017). It has been proposed that a high dietary intake of seafood and other sources of long-chain omega-3 polyunsaturated fats (LC-n3-FA) may have long-term beneficial effects on cognitive function (Hardman et al., 2016). The incorporation of fish-based nutrition in one’s diet has been shown to maintain gray matter volumes in the hippocampus, precuneus, posterior cingulate, and orbital cortex (Raji et al., 2014). The Italian Longitudinal Study on Aging found that increased daily dietary intake of olive oil is protective against age-related changes in cognitive function (Maggi et al., 1994). A systematic review reported that milk intake was inversely associated with the risk of cognitive impairment. The preventive role of a diet rich in milk may be attributed to its protein, vitamins, and essential amino acids (Wu and Sun, 2016). Our previous studies showed that dietary intake of fish, shrimp, nut, egg, vegetable, and fruit might be beneficial for the cognitive function of elderly Chinese adults (Zhao X. et al., 2015; Dong et al., 2016; Yuan et al., 2016). These studies examined the association between daily diet and cognitive function. Dietary nutrients may play an important role relating to how daily diet affects cognitive function. This prospective cohort study showed that dietary intake of riboflavin and USFA can improve multi-domain cognitive function in the middle-aged and elderly populations. These associations remained significant and consistent after we performed sensitivity analyses.

Riboflavin is found in a wide variety of animal- and plant-based foods, mostly in the form of protein-bound flavin mononucleotide (FMN) or flavin adenine dinucleotide (FAD). Riboflavin-rich foods are meat, dairy products, eggs, green leafy vegetables, and legumes. There are a number of potential causes of riboflavin deficiency including inadequate intake (for example, chronic dieters), increased requirements (for example, elderly), malabsorption (for example, gastrointestinal and biliary obstruction, renal insufficiency, diabetes, and liver disease), drug-nutrient interactions (for example, phenothiazine and theophylline), as well as others (for example, alcohol abuse, genetic disorders, and hypochromic anemia) (Porter et al., 2016). The most common cause of riboflavin deficiencies in elderly people is related to low dietary intake (Porter et al., 2016). In addition, the aging process itself can negatively correlate to the absorption, transport, and metabolism of riboflavin, and thus elderly people have increased riboflavin requirements (Porter et al., 2016). In different sensitivity analysis models, the protective effects of riboflavin on cognitive function were significant and consistent. Moreover, compared with the low-riboflavin group, the neuropsychological measure scores showed reduced declines in the high-riboflavin group. Oxidative stress is implicated as one of the primary causes of cognitive impairment. The current existing evidence indicates that riboflavin is an antioxidant nutrient, which may prevent lipid peroxidation and oxidative injury (Saedisomeolia and Ashoori, 2018). As the reduction of oxidized glutathione is a riboflavin-dependent process, riboflavin can effectively increase the level of reduced glutathione (GSH) in tissues (Alam et al., 2015; Al-Harbi et al., 2015; Chen et al., 2015a, b). A poor riboflavin status is also related to a decrease in antioxidant enzyme activity, and riboflavin administration has also been reported to enhance the activity of antioxidant enzymes (Saedisomeolia and Ashoori, 2018). Several studies have reported the effect of riboflavin on cognitive impairment (Kim et al., 2014; Araki et al., 2017). In another study, based in Japan, compared with the group that had a high dietary intake of riboflavin (≥1.11 mg/day), the incidence of cognitive impairment of elderly men in the group that had a low dietary intake of riboflavin (≤0.96 mg/day) was significantly higher (HR = 4.7, 95% CI: 1.3, 17.3) (Araki et al., 2017). Riboflavin may also have other physiological mechanisms that improve cognitive function in the middle-aged and elderly populations, with the exception for its anti-oxidation mechanism. Saedisomeolia and Ashoori (2018) reported that riboflavin partly functions to exert neuroprotective effects in some neurological disorders, for example, anti-oxidation, myelin formation, and mitochondrial function. However, the exact mechanism of how riboflavin acts as a neuroprotective factor is not yet understood. These unique physiological mechanisms of riboflavin may require further research in the future.

The metabolic profiling study suggests that dysregulation of USFA’s metabolism in the brain plays an important role in AD pathology. A non-targeted metabolomic study, based on brain tissue samples from 43 individuals whose ages ranged from 57 to 95 years old, identified significant differences in the abundance of six USFAs (linolenic acid, linoleic acid, EPA, DHA, arachidonic acid, and oleic acid) between an AD group and a control group (Snowden et al., 2017). USFA oxidation metabolites showed good predictive accuracy for the development of MCI. This suggests that USFA oxidation metabolites might be a potentially useful diagnostic biomarker for MCI (Burte et al., 2017). In different sensitivity analysis models, the protective effects of USFA on cognitive function were significant and consistent. In addition, compared with the low-USFA group, the neuropsychological measure scores showed reduced declines in the high-USFA group. The USFA composition of the brain membrane is directly associated to diet, which indicates that dietary USFA may play an important role in brain health (Moore et al., 2018). USFA can be ingested through plant derivatives that contain alpha linoleic acid (ALA), fish, and marine products that are rich in omega-3 fatty acids, or through DHA and EPA supplements (Olivera-Pueyo and Pelegrin-Valero, 2017). PUFAs, such as EPA and DHA, have potential benefits in cognitive function (Gillette-Guyonnet et al., 2013; Moore et al., 2018). USFA is essential for brain tissue development and function; moreover, it plays an important role in improving the brain antioxidant and cognitive activities (Hashimoto et al., 2017). USFA is also involved in multiple brain functions, including increased activity of membrane-bound enzymes, modification of the number and affinity of membrane receptors, modification of ion channel function, and modification of the neurotransmitter production and activity. These effects tend to favor the permeability of the neuronal membranes, favoring its activity, action capacity, and speed of response (Olivera-Pueyo and Pelegrin-Valero, 2017). In a parallel-group randomized clinical trials involving cognitively healthy volunteers from Spain, the global cognition function in the intervention group [supplementing the olive oil (1 L/week) and nuts (30 g/day), which contain large amounts of USFA] was significantly better than the control diet group (Valls-Pedret et al., 2015). These findings are consistent with the results of this study.

In addition, as part of the stratified analysis based on gender, this study found that riboflavin and USFA had better protective effects on cognitive function in the female population. Some studies showed that the absorption and metabolism of riboflavin and USFA might be different between males and females (Chuang et al., 2011; Onozato et al., 2015; Shin and Kim, 2019). The different absorption and metabolism of nutrients could result in different protective effects on cognitive function when comparing males and females. However, the underlying mechanism of a gender’s effect on nutrients is still not fully clarified; further research is required. This study showed that age and BMI are the risk factors for cognitive function regression. Many studies have shown that physical activities can help to preserve cognitive health in old age (Flicker et al., 2011; Najar et al., 2019). Furthermore, increasing evidences reveal a relationship between obesity and cognitive impairment (Cifre et al., 2018; Zhang et al., 2018). In this study, BMI was found to be a risk factor in cognitive function. Physical activity may play an important role in reducing BMI. Thus, it would be beneficial to recommend increased physical activity to obese people in order to improve cognitive function. However, as a limitation of the current study, we did not collect data relating to participant physical activity. The role of physical activity in cognitive function needs to be further explored in future studies. These results may also suggest that dietary intake of riboflavin and USFA may be more beneficial and more important in elderly individuals with high BMI.

This study was a longitudinal study, which revealed the temporal sequence of dietary nutrient intake and the change of cognitive functioning, which contributed to the strength of this study. Additionally, the sample size was relatively large, which allowed us to draw a robust conclusion. In order to avoid instability of results due to different patterns of adjustment, a sensitivity analysis was performed in this study. Therefore, the results of this study were reliable. However, this study was affected by some limitations. The source of riboflavin and USFA could have been food or nutrient supplements; nutrient supplement intake may influence the estimated riboflavin and USFA intake level in the middle-aged and elderly populations. In this study, researchers only estimated the nutrient intake from food, but did not estimate the nutrient intake from nutrient supplements. In future research, researchers should include the participants’ nutrient supplements in the survey questionnaire to avoid this limitation. Furthermore, this study was carried out from 2014 to 2017. A longer period of follow-up is still needed to observe the long-term effect of dietary riboflavin and USFA. The participants’ average age was only 58 in the research. Since cognitive impairment is not common at such an age, in the future, the relationship between age and cognitive function needs to be further studied in more elderly populations. This study did not measure participant biomarkers. In the future, the research team will further measure biomarkers in participants to explore the mechanisms of riboflavin and USFA when improving cognitive function. Neuropsychological measures of this study mostly covered global cognition, auditory memory, attention span, and working memory but did not cover other cognitive domains such as processing speed, executive functions, and language, which are affected in cognitive disorders. In the future, more cognitive domains need to be further studied.

## Conclusion

In conclusion, results of this study showed that by increasing daily dietary intake of riboflavin and USFA, multi-dimensional cognitive function among middle-aged and elderly people can be improved. These findings were consistent in different sensitivity analysis models. During follow-up, the neuropsychological measure scores showed a reduced decline in the high-riboflavin group and the high-USFA group.

## Data Availability

The data that support this analysis may be available on reasonable request from the corresponding author.

## Ethics Statement

The study protocol was in accordance with the Declaration of Helsinki and ethically approved by the Ethics Committee of Capital Medical University (2013SY35). All participants provided written informed consent for their participation in the study.

## Author Contributions

RX conceptualized and designed the study, obtained funding, and supervised data collection. LT and KL conducted the data analysis, drafted, and critically revised the manuscript. SC, HY, YA, YiW, XZ, YuW, and ZQ made substantial contributions to the acquisition of data, analysis, and interpretation of data. All authors read and approved the final manuscript.

## Conflict of Interest Statement

The authors declare that the research was conducted in the absence of any commercial or financial relationships that could be construed as a potential conflict of interest.

## Abbreviations

AD: Alzheimer’s disease
AVLT-IR: auditory verbal learning test-immediate recall
AVLT-LR: auditory verbal learning test-long recall
AVLT-SR: auditory verbal learning test-short recall
BMI: body mass index
CFCD: China Food Composition Database
DST-B: digit span test-backward
DST-F: digit span test-forward
FFQ: food frequency questionnaire
KMO: Kaiser–Meyer–Olkin
LMT: logical memory test
MCI: mild cognitive impairment
MoCA: montreal cognitive assessment
PCA: principal component analysis
USFA: unsaturated fatty acid.
